# Histamine H3R receptor activation in the dorsal striatum triggers stereotypies in a mouse model of tic disorders

**DOI:** 10.1038/tp.2016.290

**Published:** 2017-01-24

**Authors:** M Rapanelli, L Frick, V Pogorelov, H Ohtsu, H Bito, C Pittenger

**Affiliations:** 1 Department of Psychiatry, Yale University, New Haven, CT, USA; 2 Graduate School of Engineering, Tohoku University, Sendai, Japan; 3 Department of Neurochemistry, Graduate School of Medicine, The University of Tokyo, Tokyo, Japan; 4 Department of Psychology, Yale University, New Haven, CT, USA; 5 Child Study Center, Yale University, New Haven, CT, USA; 6 Interdepartental Neuroscience Program, Yale University, New Haven, CT, USA

## Abstract

Tic disorders affect ~5% of the population and are frequently comorbid with obsessive-compulsive disorder, autism, and attention deficit disorder. Histamine dysregulation has been identified as a rare genetic cause of tic disorders; mice with a knockout of the histidine decarboxylase (*Hdc*) gene represent a promising pathophysiologically grounded model. How alterations in the histamine system lead to tics and other neuropsychiatric pathology, however, remains unclear. We found elevated expression of the histamine H3 receptor in the striatum of *Hdc* knockout mice. The H3 receptor has significant basal activity even in the absence of ligand and thus may modulate striatal function in this knockout model. We probed H3R function using specific agonists. The H3 agonists R-aminomethylhistamine (RAMH) and immepip produced behavioral stereotypies in KO mice, but not in controls. H3 agonist treatment elevated intra-striatal dopamine in KO mice, but not in controls. This was associated with elevations in phosphorylation of rpS6, a sensitive marker of neural activity, in the dorsal striatum. We used a novel chemogenetic strategy to demonstrate that this dorsal striatal activity is necessary and sufficient for the development of stereotypy: when RAMH-activated cells in the dorsal striatum were chemogenetically activated (in the absence of RAMH), stereotypy was recapitulated in KO animals, and when they were silenced the ability of RAMH to produce stereotypy was blocked. These results identify the H3 receptor in the dorsal striatum as a contributor to repetitive behavioral pathology.

## Introduction

Histamine (HA) is produced by neurons of the posterior tuberomamillary nucleus of the hypothalamus (TMN); these neurons project broadly throughout the central nervous system.^1, 2^ Histamine has been linked to the regulation of the sleep–wake cycle and appetite control.^2, 3^ More recently, HA dysregulation has been associated with Tourette syndrome (TS), tic disorders and related pathology.^4, 5^ A rare mutation in the histidine decarboxylase (*Hdc*) gene, which is required for HA biosynthesis, was identified as a high-penetrance genetic cause of TS;^6, 7^ *Hdc* knockout mice constitute a pathophysiologically grounded model of this condition with etiologic, predictive, and face validity.^7, 8^ Other genetic studies have suggested that abnormalities in HA modulatory neurotransmission may contribute to tic disorders beyond the single pedigree in which the original *Hdc* mutation was originally identified.^9, 10^

Tics are seen at least transiently in up to 20% of children, and a smaller fraction of adults; TS, which consists of persistent vocal and motor tics, has a prevalence of ~0.7%.^11^ Tics are commonly comorbid with other neuropsychiatric pathology, including obsessive-compulsive disorder (OCD), attentional difficulties and autism.^12^ Their etiology, however, remains poorly understood. Dysregulation of the cortico-basal ganglia circuitry has been implicated.^13^ Pathophysiologically grounded models such as the *Hdc* knockout (*Hdc*-KO) create an opportunity to generate new insights and hypotheses for later investigation in patients. *Hdc* knockouts exhibit repetitive behavioral pathology: amphetamine-induced stereotypies are potentiated,^7^ and elevated grooming is seen after acute stress.^8^ Although such repetitive behaviors are not identical to tics, as described clinically in TS patients, they suggest recapitulation of relevant pathological changes in the model. *Hdc*-KO mice have dysregulated baseline striatal dopamine (DA) and elevated markers of neuronal activity in striatal neurons.^7, 14^

Histamine acts on four G-protein-coupled receptors, H1R-H4R. The H3R receptor is found only in the central nervous system.^1, 2^ It has high endogenous signaling activity and can thus modulate intracellular signaling even in the absence of agonist.^15^ Unlike the excitatory G_α_q-coupled H1R and G_α_s-coupled H2R receptors, H3R has classically been considered to be coupled to G_α_i and to act presynaptically to reduce neurotransmitter release—both of HA itself, and of other transmitters, including DA, GABA and glutamate.^1, 16, 17^ More recently it has been found that much of the H3R in the striatum is postsynaptic, and that it couples to intracellular signaling cascades in striatal medium spiny neurons (MSNs) in complex and cell type-specific ways.^2, 18, 19, 20, 21, 22^

Here we examine the H3R receptor in the *Hdc* KO model. We find it to be upregulated in the striatum in KO animals. H3R activation leads to repetitive behavioral pathology and to modestly elevated striatal DA levels in KO mice, but not in controls. This is associated with activation of neurons in the dorsal striatum; using a novel chemogenetic strategy, we show activation of these cells to be necessary and sufficient for the development of stereotypies. These data identify H3R in the dorsal striatum as a potentially important contributor to repetitive behavioral pathology and a potential target for pathophysiological investigation and therapeutic development in tic disorders and related conditions.

## Materials and methods

### Mice

Generation of *Hdc*-KO mice has been described previously;^23^ our mice have been backcrossed onto C57Bl/6 for >10 generations and have been recently described.^7, 8, 14^ The mice used in the Figures have been further backcrossed onto D1-DARPP32-Flag/D2-DARPP32-Myc BAC double transgenic mice,^24^ generously provided by Paul Greengard. *Hdc* genotype was determined by PCR.

Mice were housed in a temperature (23°) and humidity-controlled vivarium on a 12-h light/dark cycle. Two- to 3-month-old male and female mice were used in all experiments; sex was examined as an independent variable in all analyses but did not significantly affect any measured effects and thus is not reported. Experimental procedures and animal care were approved by the Yale University Institutional Animal Care and Use Committee.

### Drugs

Clozapine n-oxide (Tocris, Bristol, UK) was dissolved at 1 mg ml^−1^ in sterile saline. Salvinorin B (SalB; Cayman Chemical, Ann Arbor, MI, USA) was suspended in DMSO (25 mg ml^−1^) and then diluted to 5 mg ml^−1^ in sunflower oil (Sigma, St Louis, MO, USA). 4-OH-Tamoxifen (Sigma) was dissolved in DMSO (20 mg ml^−1^) and then diluted to 8 mg ml^−1^ in sunflower oil (Sigma). R-amino-methylhistamine (RAMH, Tocris), Immepip (Tocris) and JNJ JNJ5207852 (Tocris) were dissolved at 9 mg ml^−1^ in sterile saline. All drugs were diluted such that mice received 0.05 ml per 10 g for each drug, for all experiments.

### Binding assays and *in situ* hybridization

Mice were killed and their brains quickly dissected out, frozen on dry ice and stored at −70 °C. Brains were coronally sectioned at 20 μm on a cryostat. Slices were mounted on subbed slides and stored at −70 °C until use.

Radioligand binding was performed as previously described.^7^ Slides were preincubated at room temperature (RT) in binding buffer (50 mm Tris-HCl, pH 7.4; 120 mm NaCl; 5 mm KCl; 2 mm CaCl_2_; 1 mm MgCl_2_), and then for 60 min at RT in the same buffer with radioligand. Radioligands were: for H2 receptor, 0.1 nm ^125^I-iodoaminopontidine (Perkin Elmer, Waltham, MA, USA), with or without 3 μM tiotidine (Tocris as a specificity control); for H3 receptor, 4 nM ^3^H-N-α-methylhistamine (Perkin Elmer), with or without 4 μM promethazine (Tocris). Slides were then rinsed several times in ice-cold binding buffer, dried, and exposed to high-sensitivity film (Hyperfilm, GE Biosciences, Marlborough, MA, USA). Images were captured using a computer-controlled digital camera (Cohu, San Diego, CA, USA) and imported into ImageJ (NIH, Bethesda, MD, USA) for densitometric analysis. Each probe and the corresponding negative control were developed and processed in parallel.

*In situ* hybridization was performed as previously described.^25^ Briefly, probes were generated by *in vivo* transcription from PCR-generated templates. Primers for probe template generation were (underlined sequence corresponds to T7): H2 forward, 5′-GCCACCAAGGCCAAGAAGTG-3′ H2 reverse+T7 5′-CCAAGCCTTCTAATACGACTCACTATAGGGAGATCACCAGGAGGCCAAGAAGC-3′ H2 forward+T7, 5′-CCAAGCCTTCTAATACGACTCACTATAGGGAGAGCCACCAAGGCCAAGAAGTG-3′ H2 reverse 5′-TCACCAGGAGGCCAAGAAGC-3′ H3 forward 5′-GACGGGCTGTTCGG AAGATG-3′ H3 reverse+T7, 5′- CCAAGCCTTCTAATACGACTCACTATAGGGAGAATCCAGCCGAAGACGAGTGC-3′ H2 forward+T7, 5′-CCAAGCCTTCTAATACGACTCACTATAGGGAGAGACGGGCTGTTCGGAAGATG-3′ and H3 reverse, 5′-ATCCAGCCGAAGACGAGTGC-3′. Probe template PCR products were confirmed by sequencing. Probes were radiolabeled using ^35^S-rCTP (Perkin Elmer), using the MAXIscript T7 polymerase kit (Ambion, Foster City, CA, USA), following the manufacturer’s instructions, and purified using a mini-QuickSpin RNA column (Roche, Indianapolis, IN, USA).

Slices were post-fixed 10 min using 4% paraformaldehyde in saline. Fixed slices were incubated overnight at 55 °C with hybridization buffer (50% v/v formamide, 3 × SSC, 50 mm NaPO_4,_ 10 mm dithiothreitol, 1 × Denhardt’s solution, 0.25 mg ml^−1^ tRNA, 10% dextran-SO_4_) containing radiolabeled sense or antisense probe for H2 or H3 receptor mRNA. Slides were then washed at RT several times with increasingly dilute SSC, dH_2_O, and 100% ethanol, air dried for several hours, and then exposed to autoradiographic film (Hyperfilm, GE Biosciences). Developed film was captured using a computer-controlled digital camera (Cohu) and imported into ImageJ (NIH) for densitometric analysis. Each probe and the corresponding sense control were developed and quantified in parallel.

### Behavioral analysis

For piloting RAMH doses, saline or RAMH was injected intraperitoneally (IP). Mice were videotaped for 30 min using an automated system (HomeCageScan; CleverSys, Reston, VA, USA). Stereotypy was quantified from videotape by an observer (VP) blind to condition, as previously described.^7^ The stereotypic behaviors expressed and considered for this kind of analysis were: sniffing, focused sniffing and licking/shaking. These observations were corroborated by measuring stereotypical beam-breaks with an automated system, as described below.

For locomotor activity monitoring, animals were habituated to the behavioral room for 60 min and then placed in an activity monitoring apparatus 47 cm L × 36.8 cm W × 20 cm H, Omnitech Electronics, Columbus, OH, USA) for 30 min before recording. Mice were injected with RAMH or saline, and/or other drugs 10 min into this period (that is, 20 min before starting recording). Activity was then recorded for 1 h. Ambulatory activity was defined as sequential infrared beam-breaks during locomotion. Stereotypical beam-breaks were defined as repetitive breaks of a single infrared beam within one second.^26, 27, 28, 29^ Beam-break data were collected and analyzed using Fusion software (Omnitech Electronics).

### *In vivo* microdialysis

Microdialysis was performed as described previously.^14^ Mice were surgically implanted with guide cannulae targeting the dorsal striatum (AP +0.5 mm, ML 2.0 mm, DV −2.2 mm) under ketamine/xylazine anesthesia using standard stereotaxic technique, with reference to the atlas of Paxinos.^30^ Guide cannulae were affixed to the skull using bilateral skull screws and Cerebond skull fixture adhesive (Plastics One, Roanoke, VA, USA). Dummy cannulae were inserted into the guide cannulae during recovery to ensure patency.

Following 3–5 days recovery, a microdialysis probe (1 mm CMA-7, 6 kDa cutoff; CMA Microdialysis, Stockholm, Sweden) was inserted through the guide and mice were left in the home cage for 20–24 h to habituate to this microdialysis cannula. The next day, with mice in their home cage, the cannula was connected to a 2.5 ml Hamilton syringe and continuously perfused with artificial cerebrospinal fluid (aCSF; Harvard Apparatus, Holliston, MA, USA) at a rate of 2 μl/min using a programmable infusion pump (CMA Microdialysis). Dialysate was collected in 10 min per 20 μl fractions on ice and stored at −70 °C for later analysis. After microdialysis a small amount of toluidine blue dye was injected through the cannula; mice where then killed and their brains removed, sliced and examined to confirm cannula placement. All cannulae were successfully targeted to the dorsal striatum, as intended. DA was measured using HPLC with electrochemical detection as described previously.^7^

### Molecular characterization of striatal activity

*Hdc*-KO and WT mice were injected with saline or RAMH (45 mg kg^−1^) and transcardially perfused 30 min later with 4% paraformaldehyde in 1 × PBS supplemented with 0.1 mm NaF. Brains were stored at −80° until slicing.

Brains were sliced on a cryostat at 30 μm; slices were stored in a solution containing 30% glycerin, 30% ethylene glycol and 1 × tris-buffered saline (TBS) plus 0.1 mm NaF. Slides were washed 3 × 10 min in TBS, incubated for 1 h at RT in TBS with 0.2% Triton X-100 and 5% donkey serum (Jackson Immnoresearch, West Grove, PA, USA), and then incubated overnight at RT in the same solution with rabbit anti-phospho-rpS6 235/236 (#4858S, 1:500, Cell Signalling Technologies, Beverly, MA, USA). Slices were then rinsed 3 × 10 min at RT in TBS with 0.2% Triton X-100 and 5% donkey serum, and then incubated for 1 h at RT in the same buffer with Alexa Fluor 555 donkey anti-rabbit (Life Technologies, A31572, 1:500). Slices were then rinsed in TBS, mounted on subbed slides, cover-slipped, and stored at 4 °C.

Confocal imaging was performed by sequential scanning at 20 × using an Olympus Fluoview FV-1000 confocal microscope equipped with 473, 559 and 635 nm lasers. Images were acquired with a Kalman filter at a scan rate of 4 μs per pixel. 20 μm Z-stacks were collected with a step size of 0.2 μm. Quantification was performed as was previously described.^22^ One image was taken from the dorsal striatum of each hemisphere of each slice, to avoid overlap. Quantification was performed by counting positive cells above background throughout each Z-stack. All clearly stained cells in each Z-stack were categorized and quantified; immunopositive cells that could not be unambiguously categorized as a positive cell were excluded. The total number of cells was averaged for each mouse for analysis; *N* for each experiment thus reflects the number of mice, not of slices.

### Viral infusions and activity-dependent cell tagging with DREADDs

Viral infusions into the dorsal striatum were performed as previously described.^31^ D1/D2 *Hdc* KO and D1/D2 *Hdc* WT mice were anesthetized with ketamine/xylazine (100/10 mg kg^−1^) and placed in a stereotaxic frame. Viral infusion was performed bilaterally into the striatum (AP +0.5, ML±1.95, DV −2.7) using a 1 μl Hamilton syringe attached to a micropump (UltraMicroPump II, WPI). A volume of 0.5 μl was injected into each hemisphere at a flow rate of 0.1 μl min^−1^.

Three viruses were infused together: AAV2-E-SARE-ER^T2^-CreER^T2^-PEST^32^ (10^11^ viral particles, produced as described in ^33^); AAV8-hSyn-DIO-HA-KORD(Gi)-IRES-mCitrine^34^ (10^12^ viral particles, produced by UNC Vector Core (www.med.unc.edu/genetherapy/vectorcore)); and AAV5-hSyn-DIO-hM3D(Gq)-mCherry^35^ (10^12^ viral particles, produced by the UNC Vector Core). Activity-dependent cell tagging was achieved using the virus AAV-E-SARE-ER^T2^-CreER^T2^-PEST, which expresses tamoxifen-regulated Cre recombinase in active cells, under control of the synthetic activity-dependent E-SARE promoter.^32^ This is similar to the activity-dependent Fos promoter used in previous studies,^36, 37, 38^ but the E-SARE promoter has a substantially improved signal-to-noise ratio, and the use of two ER^T2^ domains in the ER^T2^CreER^T2^ double fusion protein provides a dramatic reduction in leakiness relative to similar ligand-gated recombinase systems.^32, 39^ Furthermore, this addition of the PEST sequence to this fusion protein leads to a reduced half-life^39^, narrowing the recombination window and further reducing background.

Two weeks after virus infusion, mice were injected IP with 4-OH-tamoxifen together with either saline or 45 mg kg^−1^ RAMH, to allow activity-dependent cell tagging through recombination of KORD and hM3D designer receptors exclusively activated by designer drugs (DREADD) viruses in active cells of the dorsal striatum. Behavioral analysis was initiated one week after cell tagging.

### Confirmation of DREADD expression

Brains were fixed by transcardiac perfusion, extracted, sliced, and immunostained as described above. Immunostaining was with chicken anti GFP (Abcam ab13970 1:1000), goat anti-Myc (Abcam ab9132 1:500), mouse anti-mCherry (Abcam, 125096, 1:200) and rabbit anti HA (Cell Signaling Technologies, C29F4, 1:500). Secondary detection was performed with: Alexa Fluor 633 donkey anti-goat secondary (Life Technologies, A21082, 1:500), Alexa Fluor 555 donkey anti-rabbit secondary (Life Technologies, A31572 1:500), Alexa Fluor 488 donkey anti chicken and Alexa Fluor 488 donkey anti-mouse (Life Technologies, A21202 1:500).

### Statistical analysis

All values are expressed as mean±s.e.m. Statistical analyses were performed using GraphPad Prism using parametric analysis of variances (ANOVAs) followed by Sidak’s *post hoc* test, except for three-way ANOVA analyses described in Supplementary Figure 2, which were performed using SPSS (IBM, Armonk, NY, USA) or Minitab. All comparisons were considered significant at *P*<0.05.

## Results

### Altered H2R and H3R binding in the striatum of *Hdc*-KO mice

*Hdc-*KO mice have been shown to have alterations in HA receptor expression in hippocampus and TMN;^40^ HA receptor expression has not previously been examined in the basal ganglia in these mice. We used both radioligand binding and *in situ* hybridization to examine H2 and H3 HA receptors, which have been shown to modulate striatal function.^16^

**Figure 1:**
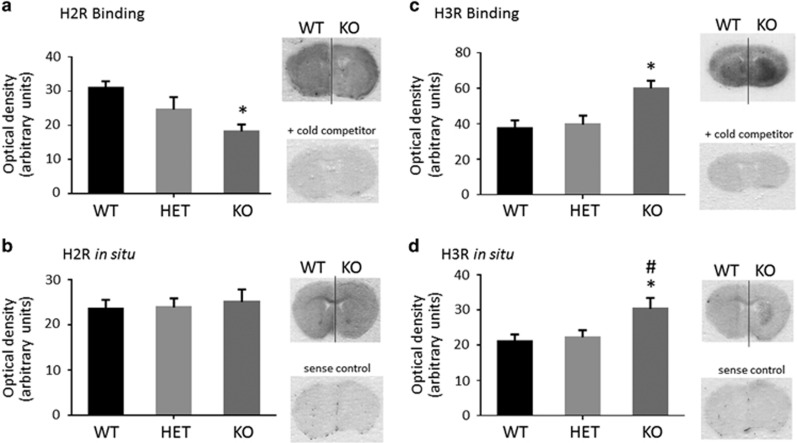
H2R and H3R in *Hdc*-KO mice. (**a**). H2R in *Hdc-*KO mice. Binding with the specific ligand ^125^I-iodoaminopontidine was reduced in the striatum in KO mice, and intermediate in heterozygotes (one-way analysis of variance (ANOVA): F[2,15]=5.16, *P*=0.015). (**b**) There was no detectable genotype effect on *H2R* mRNA in *Hdc-*KO mice (one-way ANOVA: F[2,15]=0.12, *P*>0.8). (**c**). H3R in *Hdc*-KO mice. Binding with the specific ligand ^3^H-*N*-α-methylhistamine was increased in the striatum in *Hdc*-KO mice (one-way ANOVA: F[2,15]=7.2,=0.006). (**d**). The same effect was seen on *Hdc* mRNA (one-way ANOVA: F[2,15]=4.3, *P*=0.034). *N*=6 WT, *N*=6 *Hdc* heterozygous, *N*=6 *Hdc*-KO; *—Sidak’s *post hoc*, *P*<0.05 relative to WT; ^#^*P*<0.05 relative to HET.

H2R receptor mRNA was not altered in the striatum of *Hdc*-KO or *Hdc*-heterozygous mice, but H2R radioligand binding was reduced in a gene dose-dependent manner (Figures 1a and b). H3R, on the other hand, was increased in *Hdc*-KO mice, as measured both by radioligand binding and by *in situ* hybridization (Figures 1c and d).

### H3R activation produces stereotypy in *Hdc-*KO mice

*Hdc*-KO mice have undetectable levels of HA in the striatum;^7^ but the H3 receptor has high constitutive activity^15^ and thus may modulate striatal function in *Hdc*-KO mice even in the absence of its endogenous ligand. We tested the effects of H3R activity in these mice by administering the agonist R-amino-methylhistamine (RAMH). RAMH has no affinity for H1R and H2R and 200-fold higher affinity for H3R over H4R.

First, in a dose-finding study, we injected increasing doses of RAMH and measured locomotor activity and stereotypy, scored from video (Supplementary Figure 1). Stereotypy was increased by RAMH in KO mice, in a dose-dependent manner.

**Figure 2:**
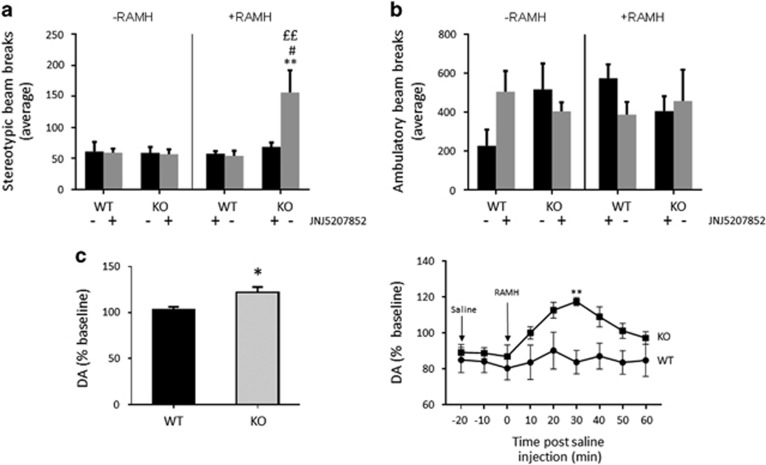
Effects of H3 activation on behavior and striatal dopamine in *Hdc*-KO mice. (**a**) Stereotypic beam-breaks in an open field were elevated by RAMH in *Hdc*-KO mice, but not in wild-type controls; this was attenuated by the H3R antagonist JNJ5207852 (three-way analysis of variance (ANOVA): main effect of genotype, F[1,38]=4.85, *P*=0.036; main effect of RAMH, F[1,38]=5.09, *P*=0.032; main effect of JNJ5207852, F[1,38]=3.11, *P*=0.88; interaction RAMH × genotype, F[1,38]=6.95, *P*=0.013; interaction genotype × JNJ5207852, F[1,38]=3.38, *P*=0.076; interaction RAMH × JNJ5207852, F[1,38]=3.24, *P*=0.082; interaction genotype × RAMH × JNJ5207852, F[1,38]=4.519, *P*=0.0413. p-values corrected for FDR (Benjamini and Hochberg), **P<0.01 HDC-KO_RAMH_ vs WT_saline_; ^#^*P*<0.05 HDC-KO_RAMH_ vs HDC-KO_RAMH+JNJ5207852_; ££ *P*<0.01 HDC-KO_saline_ vs HDC-KO_RAMH_. (**b**). Ambulatory beam-breaks, in contrast, did not differ between groups; there was a trend towards elevated ambulatory count in KO mice, irrespective of H3 agonist treatment, but it did not reach significance (three-way ANOVA: no significant main effects or interactions. *N*=6 WT_saline,_ *N*=4 HDC-KO_saline_, *N*=6 WT_RAMH_, *N*=4 HDC-KO_RAMH_, *N*=5 WT_RAMH+JNJ5207852_, *N*=5 HDC-KO_RAMH+JNJ5207852_. (**c**). RAMH challenge led to a modest but significant elevation in striatal DA in KO but not in WT mice. Left, average DA, normalized to baseline, across 60 min after RAMH or saline injection: *t*[10]=3.1, *P*=0.011. Right, time course following RAMH challenge, RM-ANOVA: main effect of genotype, F[1,10]=9.7, *P*=0.011; main effect of time, F[8,80]=6.45, *P*<0.0001; interaction, F[8,80]=3.8, *P*<0.001). *N*=7 WT, *N*=5 KO. Sidak’s *post hoc*: **P<0.05. ANOVA, analysis of variance; RAMH, R-aminomethylhistamine.

We replicated this stereotypy effect in a larger cohort of animals with the highest dose of RAMH (45 mg/kg, intraperitoneal (i.p.)), using beam-breaks during exploration as an automated measure of stereotypy (defined as repetitive breaking of a single beam, which indicates repetitive low-amplitude movement).^26, 27, 28, 29^ Stereotypic counts were strongly potentiated by RAMH in *Hdc-*KO animals (Figure 2a). This was completely abolished when RAMH was administered together with the H3R antagonist JNJ5207852 (10 mg kg^−^^1^, i.p.). Ambulatory beam-break counts (defined as breaking of sequential beams) were unchanged (Figure 2b).

We confirmed the effect of H3R activation using the H3R agonist immepip (20 mg kg^−1^, i.p.). Like RAMH, immepip-induced stereotypic beam-breaks only in HDC-KO mice (Supplementary Figure 2). The effects of immepip on the HDC-KO mice were completely blocked by JNJ5207852 (Supplementary Figure 2).

HA regulates striatal DA.^7^ H3R has been shown to negatively regulate DA release *ex vivo*,^17^ and we speculated that it might have a similar effect *in vivo* in the striatum. Contrary to this prediction, however, RAMH challenge had no measurable effect on striatal DA concentration, measured by *in vivo* microdialysis, in WT mice, and led to a modest but statistically significant elevation in striatal DA in *Hdc*-KO animals (Figure 2c).

### Dorsal striatal activation by H3R activation

**Figure 3:**
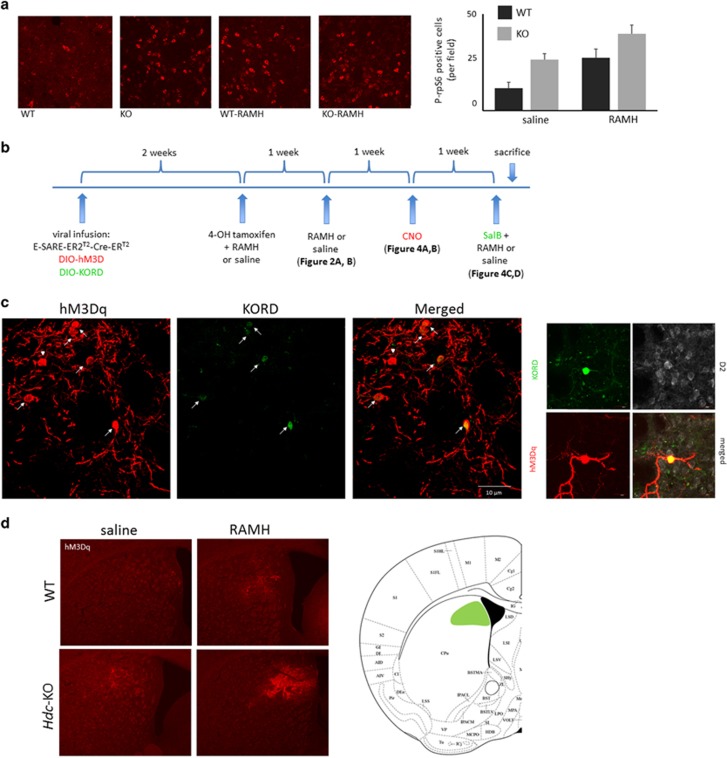
DREADD tagging of RAMH-activated cells in dorsal striatum. (**a**) RAMH leads to cell activation in the dorsal striatum. Representative confocal images of immunostaining for P-S235/236-rpS6 in the dorsal striatum are shown for each condition. Immunopositive cells from Z-stacks through the dorsal striatum were counted, blind to experimental condition; there was a main effect of both genotype and RAMH. *N*=8 WT_saline_, *N*=8 WT_RAMH_, *N*=8 HDC-KO_saline_, *N*=8 HDC-KO_RAMH_. 2 × 2 analysis of variance: main effect of genotype, F[1,28]=15.7, *P*=0.0005; main effect of treatment, F[1,28]=13.8, *P*=0.0009; interaction, F[1,28]=0.10, *P*=0.75. (**b**) Strategy for dual tagging of activated dorsal striatal cells and subsequent behavioral testing. Tagging of activated dorsal striatal cells for subsequent chemogenetic regulation was accomplished by injection of virus expressing TMX-activated Cre recombinase under the activity-regulated E-SARE promoter together with Cre-activated viruses expressing the hM3D and KORD DREADDs. (**c**) Confocal imaging documented co-expression of hM3Dq and KORD DREADDs (left, × 40); higher-magnification of a single cell, together with immunostaining for the Myc tag that is expressed in D2 neurons in these mice (right, × 60). (**d**) Low-magnification fluorescent images of hM3Dq DREADD document typical spread and level of expression in the dorsal striatum in all experimental groups (× 4 magnification). A composite depiction of viral spread in all animals in the KO-RAMH group, which shows the highest expression of the DREADD receptor and is the critical group for subsequent behavioral analysis, is shown. DREADD, designer receptors exclusively activated by designer drugs; RAMH, R-aminomethylhistamine.

We have previously shown *Hdc*-KO mice to have elevated markers of neural activity in the dorsal striatum.^7, 14^ We examined the effect of RAMH on the phosphorylation of riboprotein S6 (rpS6), which is sensitively regulated by neural activity in the striatum.^41^ Cells immunopositive for rpS6 were counted in confocal Z-stacks, blind to experimental condition, in WT and KO mice killed 30 min after injection of saline or RAMH (45 mg kg^−1^, i.p.). Phosphorylation of rpS6 was increased at baseline in *Hdc*-KO mice, consistent with previous results;^14^ rpS6 phosphorylation was increased by RAMH treatment in both WT and KO animals (Figure 3a).

### Necessity and sufficiency of dorsal striatal neuronal activity for RAMH-induced stereotypy in *Hdc*-KO animals

RAMH induces repetitive behavioral pathology (Figure 2) and changes in activity-dependent signaling in the dorsal striatum (Figure 3a) in the *Hdc*-KO model of tic disorders. To test the causal relationship between these observations we developed a novel strategy for the tagging and bidirectional chemogenetic regulation of RAMH-activated cells in the dorsal striatum (Figure 3b). An inducible Cre virus, AAV2-E-SARE-ER^T2^-CreER^T2^-PEST,^32^ was co-infused into the dorsal striatum with two Cre-activated DREADD-expressing viruses: AAV-hSyn-DIO-hM3D(Gq)-mCherry, which allows the chemogenetic activation of tagged cells through systemic administration of clozapine N-oxide (CNO),^35^ and AAV-hSyn-DIO-HA-KORD(Gi)-IRES-mCitrine, which allows the chemogenetic silencing of tagged cells through systemic administration of salvinorin B (SalB). DREADDs have previously been shown to effectively and bidirectionally regulate MSN activity.^37^

Two weeks following surgery, mice were injected with 4-OH-tamoxifen (TMX, 50 mg kg^−1^, i.p.) together with either saline or 45 mg/kg RAMH (i.p.). This leads to Cre-mediated recombination, and thus activation, of both DREADD viruses, but only in the dorsal striatum, and only in cells that are activated after RAMH (or saline) administration (Figures 3c and d). Co-expression of both DREADDs was seen in cells in the striatum, with low levels of singly tagged cells (Figure 3c), consistent with previous results in other cell types.^34^ This produced four groups of mice: WT mice in which TMX was paired with saline (hereafter WT_saline_), WTs in which TMX was paired with RAMH (WT_RAMH_), *Hdc*-KO mice in which TMX was paired with saline (*Hdc*-KO_saline_), and *Hdc*-KO mice in which TMX was paired with RAMH (*Hdc*-KO_RAMH_). Response of these mice to RAMH or saline, with no DREADD activation, is shown in Figures 2a and b.

**Figure 4:**
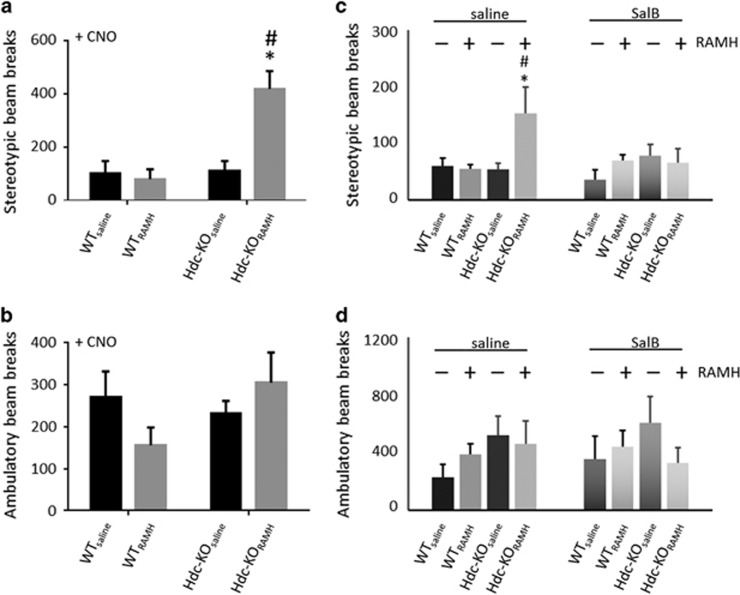
Chemogenetic testing of the sufficiency and necessity of dorsal striatal neuronal activity for stereotypic behavior. (**a**) CNO injection produced elevated stereotypic beam-breaks in *Hdc*-KO_RAMH_ mice but not in other experimental groups. Two-way analysis of variance (ANOVA): main effect of genotype F[1,18]=13.5, *P*=0.0018; main effect of previous RAMH/saline, F[1.18]=9.1, *P*=0.0074; interaction, F[1,18]=12.1, *P*=0.0027. Sidak’s *post hoc*: *P<0.05 vs WT_RAMH_; ^#^*P*<0.05 vs HDC-KO_saline_; *N*=6 WT_saline_, *N*=6 WT_RAMH_, *N*=5 HDC-KO_saline_, *N*=6 HDC-KO_RAMH_. (**b**) CNO did not significantly affect ambulatory counts in any group. Two-way ANOVA: main effect of genotype, F[1,18]=1.04, *P*=0.32; main effect of previous RAMH/saline, F[1,18]=0.15, *P*>0.7; interaction, F[1,18]=3.04, *P*=0.098; *N*=6 WT_saline_, *N*=6 WT_RAMH_, *N*=5 HDC-KO_saline_, *N*=6 HDC-KO_RAMH_. (**c**) SalB completely blocked the elevation in stereotypy seen after RAMH in *Hdc*-KO mice (saline data are re-plotted from Figure 2a). *N*=6 WT_saline_, *N*=6 WT_RAMH_, *N*=5 HDC-KO_saline_, *N*=6 HDC-KO_RAMH_. 3-way ANOVA: main effect of genotype, F[1,34]=5.69, *P*=0.023; main effect of RAMH, F[1,34]=4.58, *P*=0.04; genotype × RAMH × SalB: F[1,34]=7.84, *P*=0.008; other effects and interactions not significant. See Figure 2a for lower-order analysis of saline data only; the interaction between genotype and RAMH was lost after SalB treatment (no significant main effects or interactions in a 2-way ANOVA). A *post hoc* lower-order ANOVA within the *Hdc-*KO mice confirmed that SalB modulated the effect of RAMH (two-way ANOVA: SalB × RAMH interaction, F[1,14]=4.1, *P*=0.063). (**d**) RAMH has no effect on ambulatory counts in either genotype in the presence of SalB (three-way ANOVA: no significant main effects or interactions). *N*=6 WT_saline_, *N*=6 WT_RAMH_, *N*=5 HDC-KO_saline_, *N*=6 HDC-KO_RAMH_. RAMH, R-aminomethylhistamine.

To test the sufficiency of activity in RAMH-activated dorsal striatal cells to produce repetitive behavioral pathology, we injected all mice IP with clozapine-N-oxide (CNO, 5 mg kg^−1^, i.p.), the ligand of the activating hM3Dq DREADD channel, which produces burst firing in neurons.^35, 42^ This produced robust stereotypy in *Hdc*-KO_RAMH_ mice, but none in the other groups (Figure 4a). There was no significant effect of CNO on ambulatory activity in any group (Figure 4b).

To test the necessity of activity in dorsal striatum for RAMH-induced stereotypy in *Hdc*-KO mice, we administered RAMH (45 mg kg^−1^, i.p.) or saline, as in Figure 2a, together with SalB (5 mg kg^−1^, subcutaneously). Activation of the G_i_ coupled KORD DREADD by SalB blocked the stereotypical behaviors induced by RAMH (Figure 4c). Neither RAMH nor SalB affected ambulatory counts, in either genotype (Figure 4d).

## Discussion

Tic disorders are common and are commonly comorbid with a wide range of other neuropsychiatric symptoms, but their pathophysiology remains poorly understood. Alterations in the brain’s histamine modulatory system have been implicated as a contributor to the development of tics and of TS by a series of genetic studies.^6, 9, 10^ The *Hdc* knockout mouse recapitulates a rare but high-penetrance genetic cause of TS and constitutes a promising model of its pathophysiology.^6, 7^ *Hdc*-KO mice exhibit repetitive behavioral pathology, DA dysregulation, and altered markers of cellular activity and intracellular signaling in the striatum.^7, 14, 43, 44^

We have examined HA receptors in these mice and found the H3 receptor to be upregulated in the striatum (Figure 1). H3R has also been reported to be elevated in the TMN, but reduced in the hippocampus, in *Hdc*-KO mice.^40^ *Hdc*-KO mice have undetectable levels of HA in the striatum,^7^ but H3R has unusually high basal activity and thus may modulate brain function even in the absence of ligand.^15^

### Localization and functions of the H3 receptor

H3R has classically been described as a presynaptic G_α_i-coupled receptor; *ex vivo*, it can inhibit the release of HA itself as well as of DA, glutamate, and other transmitters.^1, 16, 17^ However, it is increasingly clear that H3R can also exist postsynaptically, where its signaling properties are complex and are modulated by its interactions with other cellular components, including DA receptors.^18, 19, 20, 21^ Indeed, postsynaptic signaling may be a primary function of the H3 receptor in the striatum.^2^ Distinct H3R signaling pathways in direct and indirect pathway MSNs (dMSNs and iMSNs, respectively) have been described *ex vivo*.^20^ We have recently replicated and extended this work *in vivo*, finding that H3R regulates MAPK signaling in dMSNs and Akt/GSK signaling in both dMSNs and iMSNs.^22^ These qualitatively distinct actions confirm that H3R signaling is modulated by the cell type on which the receptor is expressed.

Upregulated H3R in *Hdc*-KO mice may be functionally identical to normally expressed receptors, in which case the effects of H3R activation in the KO animals would be expected to be qualitatively identical to those in WT mice, but enhanced. Alternatively, it may be that the localization of these upregulated receptors and/or their coupling to intracellular signaling cascades is altered, compared to those in wild-type mice, and thus that the effects of RAMH are qualitatively different in the KO animals. This is an important topic for future studies in this model.

H3R agonist challenge has minimal effects in wild-type mice: RAMH challenge does not alter locomotion or stereotypy (Figure 2a and b; Supplementary Figure 1), and it does not detectably alter striatal DA (Figure 2c). Similarly, another H3R agonist, immepip, has been shown not to affect intra-striatal DA levels in wild-type mice.^45^ In *Hdc*-KO mice, on the other hand, the H3R agonist RAMH produces stereotypy in a dose-dependent manner and leads to a modest but significant elevation in striatal DA (Figure 2, Supplementary Figure 1). Similar behavioral effects are seen with the H3R agonist immepip (Supplementary Figure 2), and stereotypy after both RAMH and immepip is blocked by the H3R antagonist JNJ5207852, further confirming the specificity of these effects to H3R. To our knowledge, these data demonstrate for the first time a direct relationship between H3R activation and tic-like phenomenology, in a pathophysiologically grounded TS model.

Several considerations suggest that this effect is likely to be due to postsynaptic H3R. HA infusion reduces striatal DA,^7^ and presynaptic H3R has been described as reducing DA release *ex vivo*,^17^ though this has not to our knowledge been documented *in vivo*. But this presynaptic mechanism does not readily explain increased DA in *Hdc*-KO animals, either at baseline^7, 14^ or after RAMH stimulation (Figure 2c). On the other hand, postsynaptic H3R has been shown to modulate striatal DA: activation of H3Rs has been reported to inhibit the ability of D1R agonists to reduce striatal DA (presumably via an indirect, polysynaptic mechanism).^45^ Elevated postsynaptic H3Rs in *Hdc*-KO MSNs may be accentuating this or a related mechanism, at baseline and/or upon stimulation, and thus remove a source of inhibition of striatal DA release.

H3R activation also leads to behavioral stereotypy in *Hdc*-KO animals, but not in wild-type controls. The presence of increased DA after RAMH in these animals complicates the interpretation of this finding; but the increase in DA is so modest that it would be surprising if it alone were able to produce the repetitive behavioral pathology that we observe. As H3R activation alters phosphorylation of rpS6, a marker of MSN activity^46^, even in WT mice, in the absence of any detectable elevation in striatal DA (Figure 3), it is likely that these effects contribute to the observed repetitive behaviors, irrespective of (or in addition to) the elevation in striatal DA. Indeed, we have previously shown that H3R activation triggers rpS6 phosphorylation in wild-type mice, in a time-dependent manner.^22^ Elevated phospho-rpS6 levels in *Hdc*-KO mice at baseline may result from the constitutive activity of upregulated H3R (Figure 1).

H3R has differential effects on the direct pathway (D1-MSNs) and indirect pathway (D2-MSNs) in the striatum; in neither cell type are the effects seen *in vivo* after H3R activation well explained by canonical G_i_ coupling.^20, 22^ We speculate that constitutive H3R activity affects the balance between D1-MSN and D2-MSN activity pathway. An imbalance in these pathways in the striatum has been proposed to contribute to tics.^47, 48^ H3R agonist treatment, we propose, accentuates this imbalance, leading to stereotypic behavior. This is reflected in P-rpS6 levels after RAMH; H3R agonist treatment leads to elevated P-rpS6 in both WT and KO animals, but levels are highest in RAMH-treated KO mice. However, this interpretation remains speculative; we cannot exclude other effects of H3R, for example on interneurons and afferents.^5, 49^

### The causal importance of histamine and of dorsal striatal neuronal activity in generating repetitive behavioral pathology

Using a novel strategy to tag active neurons for chemogenetic regulation, we demonstrate that hM3Dq DREADD-mediated activation of a subset of dorsal striatal neurons—those activated after RAMH challenge in *Hdc*-KO mice—is sufficient to drive repetitive behavioral pathology, and is necessary for the development of such behavioral effects after RAMH challenge (Figure 4). The absence of any similar behavioral effects after hM3Dq DREADD activation in WT mice, or in *Hdc-*KO mice that received saline together with TMX, confirms the specificity of this activity-dependent DREADD tagging strategy.

This strategy for multiplexed chemogenetic control of a population of neurons marked by their phasic activity in response to a specific manipulation (RAMH injection, in this case) is powerful. It combines two recent technical advances. First, the E-SARE-ER^T2^-CRE-ER^T2^ construct provides optimized activity-dependent, ligand-gated cre recombinase activity, with high induction and low background.^32^ Second, the recently described G_i_-coupled inhibitory KORD DREADD,^34^ used in conjunction with the hM3Dq DREADD, allows bidirectional chemogenetic control of a cell population in the same animal. The novel combination of these technologies has potentially broad applicability for tests of the necessity and sufficiency of activity in defined neuronal populations for discrete behaviors, as we demonstrate here.

Grooming, stereotypy, and other forms of repetitive behavioral pathology have garnered increasing interest in proposed mouse models of pathophysiology associated with TS,^7, 31, 43, 50^ obsessive-compulsive disorder,^51, 52, 53, 54^ autism,^55^ Rett syndrome^56^ and other conditions.^57^ Repetitive behaviors have previously been linked to abnormalities in ventral corticostriatal circuits;^51^ the current data show the causal relevance of dysregulation of cells in the dorsal striatum, specifically, for their development.

These data particularly emphasize the potential importance of the H3 receptor for such pathology. The ability of H3R to modulate intracellular signaling irrespective of the presence of agonist^15^ makes it a potentially important regulator of basal cellular activity and signaling. It is as of yet unclear whether H3R possesses similarly high constitutive activity in its more recently appreciated modulation of postsynaptic activity of the MAPK and Akt signaling pathways in the striatum.^2, 19, 20, 22^ As HA is diurnally regulated in the striatum,^1, 7^ H3R tone is expected to be higher during the day (in humans; it is higher at night in nocturnal animals such as rodents). This may contribute to tics and other repetitive behavioral pathology being more prominent during wakefulness.

The potential relevance of the current data is clearest for TS and tic disorders, as *Hdc* mutations have been associated with TS^6, 10^ and the *Hdc*-KO mice have been characterized as a model of tic pathophysiology.^5, 7, 43^ However, some carriers of a hypomorphic *Hdc* mutation also have OCD, autism and other diagnoses, suggesting potential relevance of histaminergic pathology for these conditions as well.^6, 7^ Repetitive behavioral pathology in rodents is unlikely to map unambiguously onto existing neuropsychiatric diagnostic categories; these categories themselves have recently been critiqued.^58^ Clarifying the striatal mechanisms of repetitive behavioral pathology, and the potential contribution of H3R dysregulation to them, therefore has the potential to shed light on a range of neuropsychiatric pathology.
