# Supplementary material for: Histamine H3R receptor activation in the dorsal striatum triggers stereotypies in a mouse model of tic disorders
Source: Transl Psychiatry. 2017 Jan 24;7(1):e1013–. doi: 10.1038/tp.2016.290 (PMC5545743; doi:10.1038/tp.2016.290)
Supplement: Supplementary Figure 1 [file tp2016290x1.docx]

**
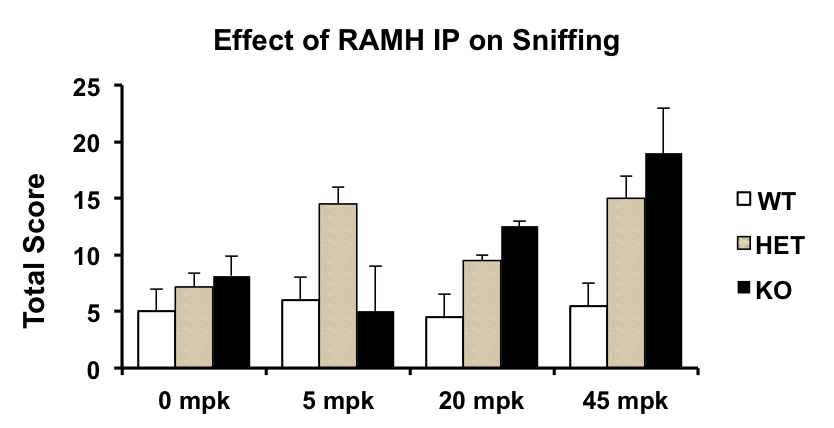

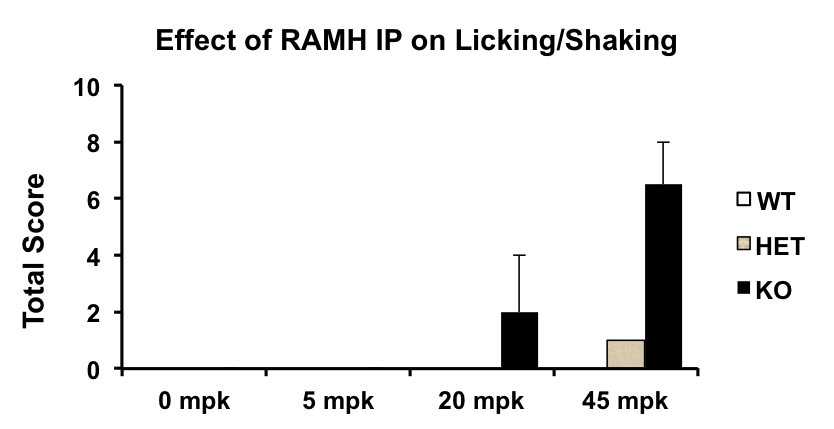

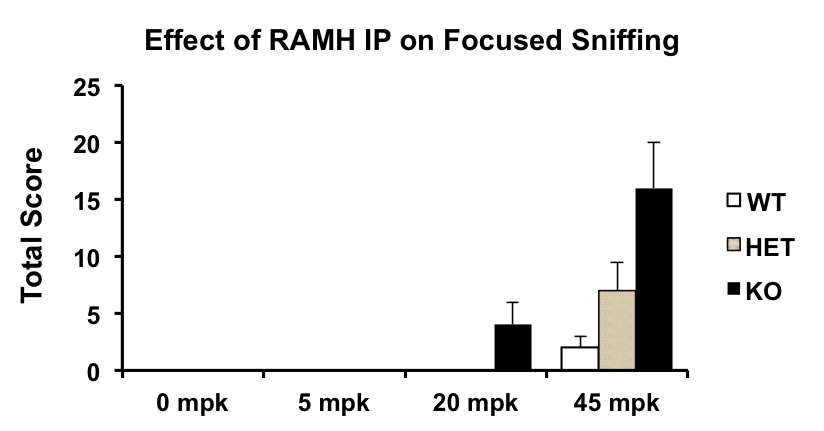

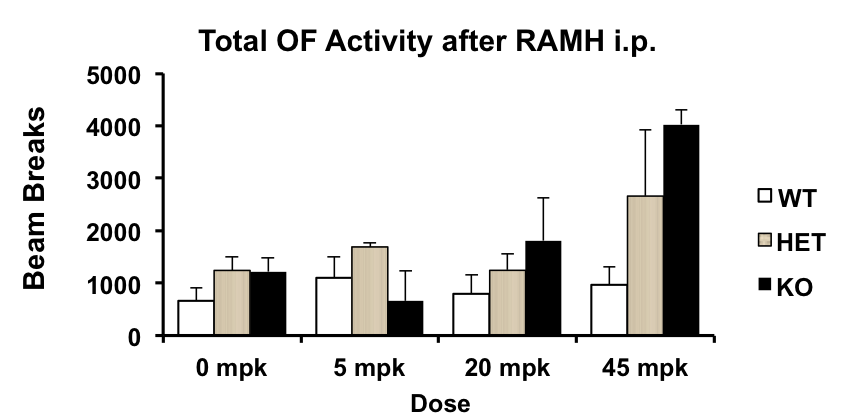
**

**D**

**C**

**B**

**A**

**Supplementary figure 1.** **Genotype-dependent effect of RAMH challenge in a dose-finding pilot experiment.** Mice (WT, *Hdc*-HET, and *Hdc*-KO) were injected with saline or with 5, 20, or 45 mg/kg RAMH and then monitored in an open field. Specific stereotypic behaviors were scored off-line from video, as described previously.^7^ The same group of animals was treated on three days, with half of the animals receiving saline on each day, to ensure that there was no drift in basal activity or stereotypy over days (there was not, and saline-treated animals are combined for analysis and presentation). **A.** Total beam-breaks increased in KO and HET mice at higher RAMH doses (ambulatory and stereotypic beam-breaks were not dissociated in this pilot experiment). 2-way ANOVA: main effect of genotype, F[2,24] = 5.7, p < 0.01; main effect of dose, F[3,24] = 6.9, p < 0.002; interaction, F[6,24] = 2.4, p = 0.06. **B.** Total sniffing, scored from video as # of discrete events during the first 20 sec of each 5 min block over 1 hour, encompasses both exploratory sniffing and stereotypic repetitive sniffing. 2-way ANOVA: main effect of genotype, F[2,24] = 7.5, p < 0.003; main effect of dose, F[3,24] = 3.87, p = 0.022; interaction, F[6,24] = 2.11, p = 0.09. **C.** Focused sniffing, which resembles the primary stereotypy we have documented in *Hdc*-KO mice after amphetamine challenge,^7^ was also scored from video as # of discrete events during the first 20 sec of each 5 min block over 1 hour observed. Focused sniffing was seen only at higher RAMH doses, and much more in *Hdc*-KO and –HET mice than in WT controls. 2-way ANOVA: main effect of genotype, F[2,24] = 5.7, p < 0.01; main effect of RAMH, F[3,24] = 12.2, p < 0.0001; interaction, F[6,24] = 3.0, p < 0.025. **D.** Stereotypic licking and body-shakes, also scored from video as # of discrete events during the first 20 sec of each 5 min block over 1 hour, was elevated at higher RAMH doses in KO *Hdc*-KO animals. 2-way ANOVA: main effect of genotype, F[2,24] = 9.9, p < 0.001; main effect of RAMH, F[3,24] = 19.3, p < 0.0001; interaction, F[6,24] = 15.6, p < 0.0001.
