# Supplementary material for: Histamine H3R receptor activation in the dorsal striatum triggers stereotypies in a mouse model of tic disorders
Source: Transl Psychiatry. 2017 Jan 24;7(1):e1013–. doi: 10.1038/tp.2016.290 (PMC5545743; doi:10.1038/tp.2016.290)
Supplement: Supplementary Figure 2 [file tp2016290x2.docx]

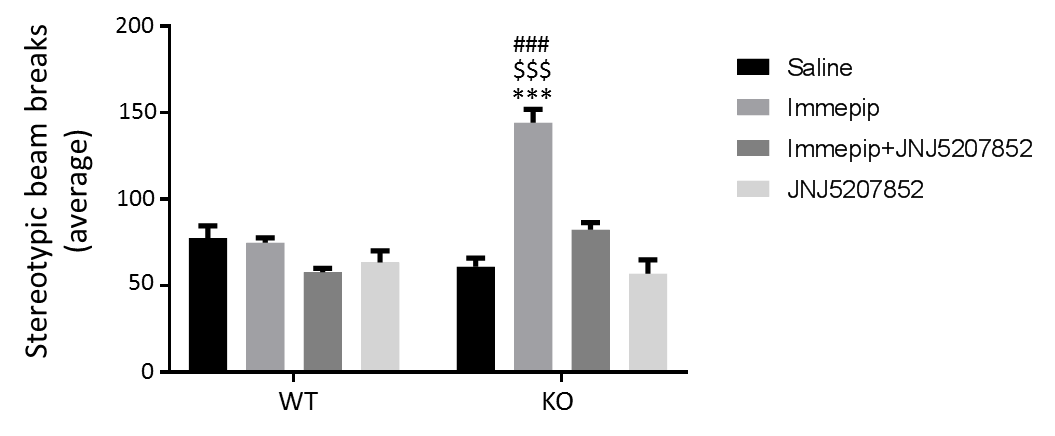


Supplemental Figure 2. H3 activation by Immepip increases stereotypic behavior in HDC-KO mice as was observed with H3 agonist RAMH. Stereotypic beam-breaks in an open field were elevated by Immepip in Hdc-KO mice, and completely abolished after JNJ5207852 (3 way-ANOVA, main effect of genotype, F[1,45] =18.53, p < 0.0001; main effect of Immepip, F[1,45] = 37.02, p < 0.0001; main effect JNJ5207852, F[1,45] = 34.71, p < 0.0001 ;genotype x Immepip interaction, F[1,45] =50.50, p < 0.0001; genotype x JNJ5207852 interaction, F[1,45] = 4.42, p = 0.042; Immepip x JNJ5207852 interaction, F[1,45] = 13.54, p = 0.001; genotype x Immepip x JNJ5207852 , F[1,45] = 11.13, p = 0.002). ***p < 0.001 HDC-KO_Immepip_ vs HDC-KO_saline_,^$$$^ p < 0.001 HDC-KO_Immepip_ vsHDC-KO_Immepip+JNJ5207852_, ^###^ p < 0.001 WT_saline_ vs HDC-KO_Immepip_. N=6 WT_saline_, N=6 HDC-KO_saline_, N=6 WT_Immepip_, N=6 HDC-KO_Immepip_, N=6 WT_Immepip+JNJ5207852_, N=6 HDC-KO_Immepip+JNJ5207852_, N=6 WT_JNJ5207852_, N=6 HDC-KO_JNJ5207852_
